# Albuminuria-Related Genetic Biomarkers: Replication and Predictive Evaluation in Individuals with and without Diabetes from the UK Biobank

**DOI:** 10.3390/ijms241311209

**Published:** 2023-07-07

**Authors:** Marisa Cañadas-Garre, Andrew T. Kunzmann, Kerry Anderson, Eoin P. Brennan, Ross Doyle, Christopher C. Patterson, Catherine Godson, Alexander P. Maxwell, Amy Jayne McKnight

**Affiliations:** 1 Molecular Epidemiology and Public Health Research Group, Centre for Public Health, Queen’s University Belfast, Institute for Clinical Sciences A, Royal Victoria Hospital, Belfast BT12 6BA, UK; 2 Genomic Oncology Area, GENYO, Centre for Genomics and Oncological Research, Pfizer-University of Granada-Andalusian Regional Government, PTS Granada, Avenida de la Ilustración 114, 18016 Granada, Spain; 3 Hematology Department, Hospital Universitario Virgen de las Nieves, Avenida de las Fuerzas Armadas 2, 18014 Granada, Spain; 4 Instituto de Investigación Biosanitaria de Granada (ibs.GRANADA), Avenida de Madrid, 15, 18012 Granada, Spain; 5 Cancer Epidemiology Research Group, Centre for Public Health, Queen’s University Belfast, Institute for Clinical Sciences A, Royal Victoria Hospital, Belfast BT12 6BA, UK; 6 UCD Diabetes Complications Research Centre, Conway Institute of Biomolecular and Biomedical Research, University College Dublin, D04 V1W8 Dublin, Ireland; 7 School of Medicine, University College Dublin, Health Sciences Centre, Belfield, D04 V1W8 Dublin, Ireland; 8 Mater Misericordiae University Hospital, Eccles St., D07 R2WY Dublin, Ireland; 9 Regional Nephrology Unit, Level 11, Belfast City Hospital, Lisburn Road, Belfast BT9 7AB, UK

**Keywords:** chronic kidney disease, diabetic kidney disease, genetic biomarkers, genetic risk score, UK Biobank, single nucleotide polymorphisms, cubilin

## Abstract

Increased albuminuria indicates underlying glomerular pathology and is associated with worse renal disease outcomes, especially in diabetic kidney disease. Many single nucleotide polymorphisms (SNPs), associated with albuminuria, could be potentially useful to construct polygenic risk scores (PRSs) for kidney disease. We investigated the diagnostic accuracy of SNPs, previously associated with albuminuria-related traits, on albuminuria and renal injury in the UK Biobank population, with a particular interest in diabetes. Multivariable logistic regression was used to evaluate the influence of 91 SNPs on urine albumin-to-creatinine ratio (UACR)-related traits and kidney damage (any pathology indicating renal injury), stratifying by diabetes. Weighted PRSs for microalbuminuria and UACR from previous studies were used to calculate the area under the receiver operating characteristic curve (AUROC). *CUBN*-rs1801239 and *DDR1*-rs116772905 were associated with all the UACR-derived phenotypes, in both the overall and non-diabetic cohorts, but not with kidney damage. Several SNPs demonstrated different effects in individuals with diabetes compared to those without. SNPs did not improve the AUROC over currently used clinical variables. Many SNPs are associated with UACR or renal injury, suggesting a role in kidney dysfunction, dependent on the presence of diabetes in some cases. However, individual SNPs or PRSs did not improve the diagnostic accuracy for albuminuria or renal injury compared to standard clinical variables.

## 1. Introduction

Chronic kidney disease (CKD) is a major global health problem with an increasing prevalence, especially in older populations [1,2,3,4]. CKD is associated with premature mortality [5] and is predicted to be among the top five causes of death worldwide by 2040 [6]. Diabetes and hypertension are common risk factors for kidney damage [7] and significant contributors to the increased CKD prevalence [3]. 

The 2012 Kidney Disease: Improving Global Outcomes (KDIGO) Clinical Practice Guideline for the Evaluation and Management of CKD defines CKD as abnormalities of kidney structure or function, present for over 3 months, with implications for health based on cause, glomerular filtration rate (GFR) category, and albuminuria category [8]. The KDIGO guidelines recommend the use of estimated glomerular filtration rate (eGFR) and albuminuria in the evaluation, classification, stratification, prognosis, and identification of the progression of CKD [8]. Higher levels of albuminuria are usually indicative of glomerular pathology and have been associated with poorer outcomes in CKD [9,10,11,12,13,14,15,16]. Albuminuria is associated with a more rapid progression of diabetic kidney disease (DKD) [17], increased risk of end-stage renal disease (ESRD) [10,12], higher risk of cardiovascular disease [11,14,16] and premature mortality [10,12,14,18,19,20]. The assessment of albuminuria is particularly relevant in the screening and diagnosis of DKD [21], as it represents an early marker of kidney pathology in individuals with diabetes and often progresses to macroalbuminuria and GFR decline [22].

The extensive search for genetic biomarkers in kidney disease, especially through Genome-Wide Association Studies (GWAS), has identified single nucleotide polymorphisms (SNPs) in genes with putative roles in urinary albumin excretion such as *UMOD*, *SHROOM3* and *ELMO1* that are strongly associated with renal diseases [23,24]. Gene variants may also be associated with serum biomarkers such as creatinine and cystatin C (and corresponding eGFR measurements), as recently reviewed [23,24]. Several GWAS have identified SNPs associated with microalbuminuria (mAlb) [25,26,27,28,29,30,31], urine albumin-to-creatinine ratio (UACR) [25,26,27,28,29,32,33] or albumin excretion rate (AER) [32,34] in CKD and/or DKD, with the most consistent findings related to rs1801239, a T/C-missense substitution in the *CUBN* gene [26,27,35,36,37], encoding the protein cubilin, involved in renal tubular albumin reabsorption [38]. Some of these SNPs have demonstrated an effect on UACR and/or albuminuria depending on the presence or absence of diabetes [26,27].

A polygenic risk score (PRS) is a number that provides an estimate of the extent to which genetic variants might influence a particular phenotypic trait. The PRS is an attempt to predict an individual’s genetic predisposition to a trait. In complex diseases, a PRS may be used for risk prediction by providing a weighted sum of contributions from risk alleles (where every single allele confers only a small effect on overall risk). Despite PRSs being increasingly used in clinical settings to improve risk prediction [39,40,41], there have been relatively few reports describing a PRS for albuminuria in renal disease [36,37]. In 2018, Pattaro et al. reviewed GWAS studies of albumin excretion, albuminuria, and proteinuria and highlighted that the data was specific to ethnic groups and usually related to DKD [36]. The same year, Haas et al. used 382,500 unrelated individuals of European ancestry in the UK Biobank (UKB) to conduct a GWAS for microalbuminuria aiming to determine if pathways increasing albuminuria are causal for cardiometabolic diseases [37]. The PRS constructed with 46 SNPs identified in the GWAS, including genes such as *CUBN* and *SHROOM3*, only explained 0.2% variance in albuminuria when validated in the Atherosclerosis Risk in Communities study and the Framingham Heart Study cohorts but was nonetheless associated with an increased risk of elevated blood pressure and hypertension [37]. It is unclear whether the SNPs identified in previous GWAS would have future applications in the prediction of albuminuria and/or UACR-related traits as part of a PRS, and in this context, the UK Biobank (UKB) provides a suitable resource to investigate the replication of those loci in a large-scale biomedical database.

The aims of this study were to replicate the effects of SNPs previously associated with albuminuria both as polygenic risk scores and as individual predictors (microalbuminuria, macroalbuminuria, UACR, or AER) [25,26,27,28,29,30,31,32,33,34] on UACR-related traits (UACR, microalbuminuria and macroalbuminuria) and investigate their influence on the development of kidney conditions indicative of renal injury in patients from the UKB cohort. We also sought to explore the effects of previously diagnosed diabetes, and to investigate the use of PRS to help improve the prediction of kidney damage.

## 2. Results

A total of 389,206 participants were included in the study, 35,806 of whom had diabetes (9.2%) (Table 1). The descriptive analysis of the main characteristics of these participants can be found in Table 1, stratified by diabetes. Ninety-one SNPs previously reported as being associated with albuminuria were included in the study (Supplementary Table S3). Among them, 16 SNPs were excluded due to MAF < 1%, HWE deviation, or LD with at least one other SNP in the set (Supplementary Table S3). 

### 2.1. Multivariable Analysis

#### 2.1.1. Association with UACR-Derived Phenotypes

**Figure 1:**
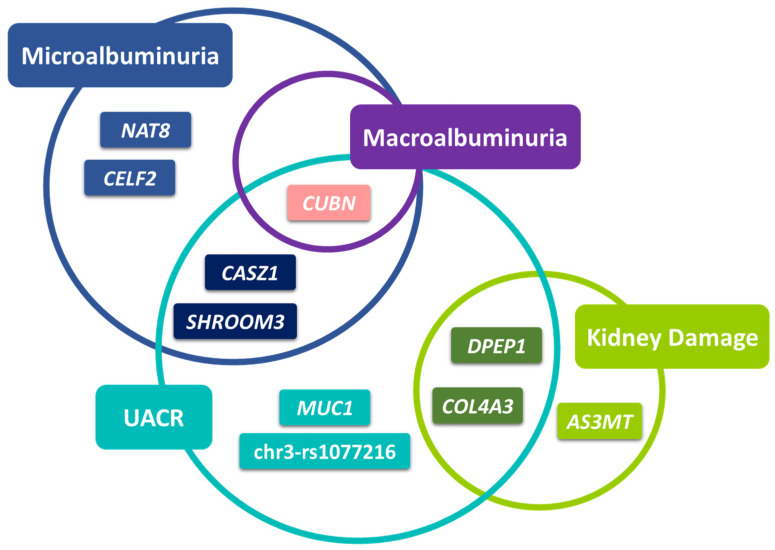
Diagram showing the genes with the most significant associations (*p* < 0.001) across phenotypes and cohorts.

As shown in Figure 1, ten SNPs showed the most significant associations across cohorts and phenotypes (*p* < 0.001). The *CUBN*-rs1801239 variant was the only one associated with all phenotypes derived from UACR. Variants in *CASZ1* and *SHROOM3* were associated with microalbuminuria and UACR, and *DPEP1* along with *COL4A3* were common for kidney damage and UACR.

**Figure 2:**
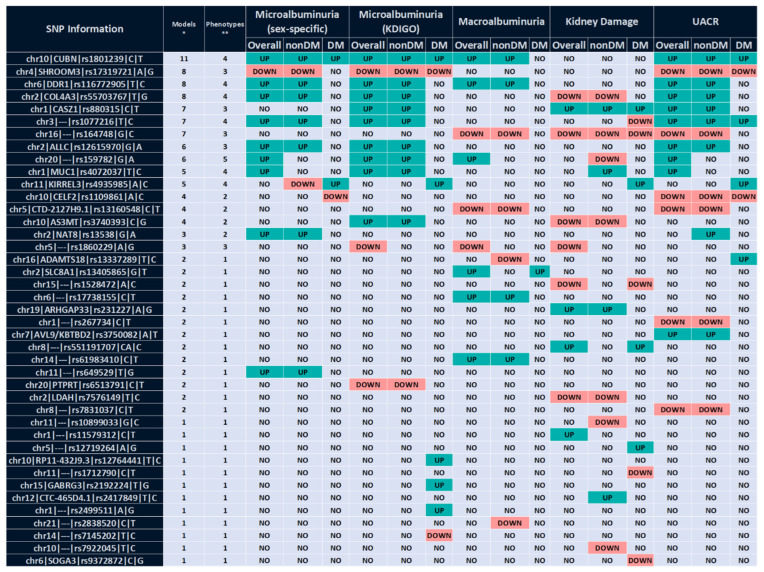
Summary of the SNPs found to be associated with the different phenotypes in each cohort. DM: diabetic cohort. KDIGO: Kidney Disease: Improving Global Outcomes. nonDM: non-diabetic cohort. SNP: single nucleotide polymorphism. UACR: urine albumin/creatinine ratio. SNP information is shown as chromosome|gene|rs identifier|counted allele|alternate allele. * Number of models including the SNP. ** Number of phenotypes associated with the SNP. Green color means the counted allele increases the beta coefficient and red is used for alleles decreasing the beta coefficient.

The results for the logistic regression of the sex-dependent and KDIGO definitions of microalbuminuria, macroalbuminuria, and kidney damage for the overall, non-diabetic, and diabetic cohorts are summarized in Figure 2.

**Figure 3:**
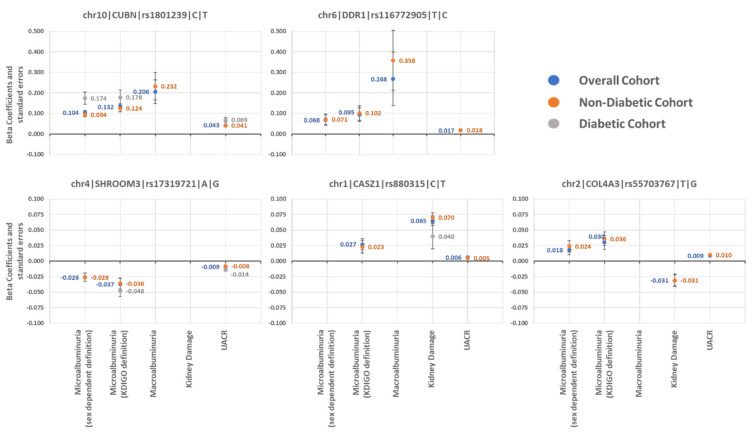
Beta coefficients and standard errors for the five single nucleotide polymorphisms with more associations across phenotypes. SNP information is shown as chromosome|gene|rs identifier|counted allele|alternate allele. The absence of dots and values means no association of the SNP with that phenotype/cohort. SNP: single nucleotide polymorphism. UACR: urine albumin-to-creatinine ratio.

Beta coefficients and standard errors for the five SNPs with more associations across phenotypes are shown in Figure 3. 

Results for every model are detailed in Supplementary Tables S4–S15. Forty-one SNPs were shown to be associated with at least one phenotype in at least one cohort. Among them, the intergenic variant rs159782 was associated with the five phenotypes in at least one of the cohorts. *CUBN*-rs1801239 and *DDR1*-rs116772905 were associated with all the UACR-derived phenotypes, (along with *SHROOM3*-rs17319721 and *ALLC*-rs12615970, except for macroalbuminuria) at least in the overall and non-diabetic cohorts, but not with kidney damage. *COL4A3*-rs55703767, rs1077216, *MUC1*-rs4072037 and *KIRREL3*-rs4935985 were associated with all phenotypes but macroalbuminuria, at least in one cohort. *CASZ1*-rs880315, rs164748 and rs1860229 were associated with three phenotypes at least in the overall cohort. Among the 12 SNPs associated with at least three phenotypes, five belonged to the PRS for microalbuminuria (*SHROOM3*-rs17319721, *DDR1*-rs116772905, rs1077216, rs159782 and *KIRREL3*-rs4935985) and four to the PRS for UACR (*CUBN*-rs1801239, *CASZ1*-rs880315, rs164748, *MUC1*-rs4072037). Despite both PRSs containing variants consistently found to be associated with several phenotypes, they did not succeed in improving the prediction significantly.

There were robust associations between SNPs and various UACR-derived phenotypes in the UKB cohort. Nevertheless, the addition of SNPs to the clinical model did not significantly improve the explanation of variance, as shown by very similar R2 values (Supplementary Tables S4–S20).

#### 2.1.2. Association with Kidney Damage

Twenty-one SNPs associated with UACR-derived variables were not associated with kidney damage, a composite phenotype of any pathology, condition or medication indicative of renal injury (Supplementary Tables S4–S12 and S16–S18), whereas 11 were specific for kidney damage (rs1528472, *ARHGAP33*-rs231227, *PRNCR1*-rs57532727, *LDAH*-rs7576149, rs10899033, *LOC105378617*-rs11579312, *CTC-465D4*.1-rs2417849, rs7922045, rs12719264, rs1712790 and *SOGA3*-rs9372872; Supplementary Tables S13–S15). 

Among the nine SNPs associated with both UACR-derived variables and kidney damage, five conserved a direction of effect consistent with the one observed in UACR-derived variables, i.e., higher risk of kidney damage with a higher risk of albuminuria or vice versa (*MUC1*-rs4072037, *KIRREL3*-rs4935985, *CASZ1*-rs880315, rs164748 and rs1860229) and four displayed opposite effects (rs159782, *COL4A3*-rs55703767, rs1077216, and *AS3MT*-rs3740393).

#### 2.1.3. Specific Association in Patients with Diabetes

Two SNPs showed different effect directions in the diabetic cohort compared to the effect in the overall or non-diabetic cohorts, *KIRREL3*-rs4935985 and *ADAMTS18*-rs13337289. The A-allele in the *KIRREL3*-rs4935985 variant was associated with higher UACR and risk of microalbuminuria and kidney damage in participants with diabetes (Supplementary Tables S6, S9, S15 and S18), but a lower risk of microalbuminuria in the absence of diabetes (Supplementary Table S5). The T-allele in the *ADAMTS18*-rs13337289 variant increased UACR in the diabetic cohort (Supplementary Table S18) but decreased the risk of macroalbuminuria in the non-diabetic cohort (Supplementary Table S11).

ROC analyses are shown in Supplementary Figures S1–S4. The inclusion of individual SNPs along with clinical variables did not improve the AUROC, as indicated by DeLong’s test for correlated curves (Supplementary Table S19). 

The results for the linear regression of UACR for the overall, non-diabetic and diabetic cohorts are shown in Supplementary Tables S16–S18. Forty-one SNPs (of the total 91 SNPs analyzed) contributed to the prediction of a phenotype in at least one of the cohorts (Figure 1).

PRS analysis can be found in the Supplementary Material (Supplementary Table S21).

## 3. Discussion

We investigated 91 SNPs previously associated with any form of albuminuria in European ancestry populations [25,26,27,28,29,30,31,32,33,34], to confirm their influence on UACR-related traits and on kidney conditions indicative of renal injury in up to 389,206 participants from the UKB cohort, representing one of the largest studies to assess the replication of SNPs associated with albuminuria or renal damage. Among the variants investigated, *CUBN*-rs1801239 was the only variant consistently showing association with all phenotypes derived from UACR with a *p* < 0.001 (Figure 1). Variants in *CASZ1* and *SHROOM3* were common for microalbuminuria and UACR, and *DPEP1* along with *COL4A3* were common for kidney damage and UACR (*p* < 0.001). Variants in *NAT8*, and *CELF2* were associated with microalbuminuria, *MUC1,* and rs1077216 with UACR and *AS3MT* with kidney damage (*p* < 0.001).

When we investigated the influence of the SNPs on UACR-derived variables and kidney damage, the addition of the SNPs as individual covariates in the multivariable model failed to add any diagnostic accuracy to the clinical models (Supplementary Figures S1–S4). Among the different SNPs analyzed, 11 were associated with at least three phenotypes in five or more models across cohorts (Figure 2), showing consistent directions of the effect with those previously reported except for *ALLC*-rs12615970 (total concordance with literature: 54%, 22 out of the total of 41 SNPs). Among them, *CUBN*-rs1801239 (*p* < 0.001), *DDR1*-rs116772905 and rs159782 showed associations with all phenotypes derived from UACR (Supplementary Tables S4–S12 and S16–S18), whereas *SHROOM3*-rs17319721 (*p* < 0.001) and *ALLC*-rs12615970 were only associated with microalbuminuria and UACR (Supplementary Tables S4–S9 and S16–S18).

*CUBN*-rs1801239 [26,27,37,42], *SHROOM3*-rs17319721 (*p* < 0.001) [27], *DDR1*-rs116772905 [28,31], *AC097662.2*-rs55703767 [28,31], *CASZ1*-rs880315 (*p* < 0.001) [26], rs1077216 [26], rs164748 [33,43], *MUC1*-rs4072037 [26], *CELF2*-rs1109861, *AS3MT*-rs3740393, rs17738155, *PTPRT*-rs6513791, *GABRG3*-rs2192224 [26], *NAT8*-rs13538, rs267734 [29], *FGF1*-rs1860229, rs2499511 [32], rs2838520 [27] and *AVL9/KBTBD2*-rs3750082 [33] showed similar effects to those previously described in the literature for any of the UACR-derived traits. 

Among the SNPs investigated in our study, 11 were specifically associated with the kidney damage phenotype, indicative of any condition of renal injury (rs1528472, *ARHGAP33*-rs231227, *PRNCR1*-rs57532727, *LDAH*-rs7576149, rs10899033, *LOC105378617*-rs11579312, *CTC-465D4*.1-rs2417849, rs7922045, rs12719264, rs1712790 and *SOGA3*-rs9372872), with the last three showing their effect specifically for participants with a previous diagnosis of diabetes (Supplementary Table S15). In particular, *ARHGAP33*-rs231227-A (Beta: 0.028; SE: 0.006; *p* = 9.5 × 10^−6^; N = 44,877) and rs2417849-T (Beta: 0.033; SE: 0.007; *p* = 4.9 × 10^−6^; N = 54,441), associated with increased UACR in individuals of European origin, predicted a higher risk of kidney damage in the overall and non-diabetic cohorts [26]. The T-allele of the rs7576149 in the *LDAH* gene was associated with decreased UACR (Beta: −0.0594; *p* = 2.2 × 10^−6^) in the GWAS discovery stage including 31,580 participants from the CKDGen Stage 1 study, although it did not show association at GWAS level in the replication or meta-analysis (*p* = 7.1 × 10^−1^ and 2.1 × 10^−4^, respectively) [27]. In our study, this allele was associated with a lower risk of kidney damage in participants from the overall and non-diabetic cohorts (Supplementary Tables S13 and S14). Similarly, the C-allele of rs10899033 was associated with increased microalbuminuria in 54,1116 individuals (Beta: 0.11; SE: 0.025 *p* = 9.3 × 10^−6^) [26], whereas the G-allele predicted lower kidney damage in our non-diabetic cohort (Supplementary Table S14). The dupA in the rs57532727 variant in *PRNCR1* was associated with ESRD when compared with macroalbuminuria (OR: 1.70; 95%CI: 1.40, 2.04; *p* = 4.4 × 10^−8^) in a GWAS meta-analysis including up to 19,406 individuals of European descent with type 1 diabetes [28], consistent with the association with kidney damage in both the overall and the diabetic cohorts in our study (Supplementary Tables S13 and S15).

In the literature, some SNPs associated with either UACR-derived or serum-derived variables (creatinine or cystatin C) have also been associated with the phenotypes CKD or ESRD. In our study, 21 SNPs showed an association with UACR-derived variables, but these were not associated with the kidney damage phenotype. Among the 20 SNPs that predicted kidney damage in any of the cohorts (Supplementary Tables S13–S15), nine were in common with any UACR variable in any cohort, although four of them (*COL4A3*-rs55703767, rs1077216, rs159782 and *AS3MT*-rs3740393) displayed the opposite effect shown by UACR-derived variables (higher risk of kidney damage despite lower albuminuria levels or vice versa).

Among the 18 SNPs associated with any trait in participants with diabetes, seven were unique to this diabetic cohort (*GABRG3*-rs2192224, rs2499511, *LOC105379144*-rs12719264, *RP11-432J9.3*-rs12764441, rs7145202, *SOGA3*-rs9372872 and *NXPE2*-rs1712790). *GABRG3*-rs2192224, rs2499511, *LOC105379144*-rs12719264 were replicated in the UKB cohort, showing consistent effects with previous results [26,32], demonstrating their potential as biomarkers for albuminuria in individuals with diabetes. The rest showed the opposite effect from that previously reported [26]. Two SNPs showed a different effect in the diabetic cohort than that displayed in the overall or non-diabetic cohort, *KIRREL3*-rs4935985 and *ADAMTS18*-rs13337289. The *KIRREL3*-rs4935985-A variant predicted higher UACR and risk of microalbuminuria and kidney damage in participants with diabetes (Supplementary Tables S6, S9, S15 and S18), whereas it predicted a lower risk of microalbuminuria in the absence of diabetes (Supplementary Table S5). The *ADAMTS18* rs13337289-T variant increased UACR in the diabetic cohort (Supplementary Table S18) while decreasing the risk of macroalbuminuria in the non-diabetic cohort (Supplementary Table S11). This variant had been previously associated with an increased risk of microalbuminuria (Beta: 0.2441; *p* = 3.2 × 10^−6^) in non-diabetic patients from the GWAS discovery stage including 31,580 participants from the CKDGen Stage 1 study, although it did not show an association at the GWAS level in the replication or meta-analysis stages (*p* = 9.7 × 10^−1^ and 1.9 × 10^−1^, respectively) [27].

In our study, neither of the calculated PRSs enhanced the diagnostic accuracy of the clinical models (Supplementary Figures S1–S4), despite these risk scores including some of the SNPs most strongly associated with clinical phenotypes, e.g., UACR (*CUBN*-rs1801239 was part of the PRS predicting UACR) and microalbuminuria (*SHROOM3*-rs17319721 and *DDR1*-rs116772905 were part of the PRS for predicting microalbuminuria). A recent study using a PRS composed of 598 SNPs associated with the main risk factors and outcomes of type 2 diabetes tested in 4098 participants from the ADVANCE study and 17,604 individuals with type 2 diabetes from the UKB has shown a precision or positive predictive value for macroalbuminuria of 19% in the top 30% high-risk group and 27% in the top 10% [44]. As in our case, the genetic PRS model did not outperform the clinical score [44]. Attempts to incorporate PRSs into the prediction of renal disease outcomes have not led to a substantial improvement over currently used risk prediction models based on clinical variables such as age, sex, albuminuria and eGFR [45,46]. The contribution of SNPs or PRSs to the prediction of renal disease is therefore marginal compared to the information provided by environmental risk factors such as cumulative glycaemic burden, age, sex, and body-mass index, among others. Since none of the PRSs tested here significantly improved the prediction of microalbuminuria or renal injury, we cannot advocate for their inclusion in patient stratification. The limited utility of PRSs is disappointing but likely reflects the small contribution of each individual risk allele to specific clinical phenotypes [47]. 

This study has several limitations, such as the challenge of utilizing PRSs in clinical risk models. SNPs and PRSs represent genetic variation; however, phenotypic diversity and disease susceptibility may be influenced by other types of variation among populations, including transcriptomic, proteomic, metabolomic, and microbiome profiles, all of which interact with diverse environmental factors [23,24,48,49]. The use of PRSs, especially when integrated with other clinical risk factors, represents a potentially useful strategy to customize healthcare and maximize clinical and public health benefits [39,40]. As recently highlighted, one of the limitations of the clinical implementation of PRSs is their transferability across different populations [50,51]. Differences in LD across ethnicities, in genetic architecture, allele frequency patterns, enrichment of homozygosity in small, bottlenecked, or highly consanguineous populations, local adaptation and epistasis due to differences in genetic backgrounds, as well as gene-environment interactions varying among populations, have been proposed as underlying factors in the failure to replicate GWAS findings across ethnic groups [50,51,52]. The scores used for the calculation of the PRSs in our work are based on GWAS for European ancestry; therefore, allelic effects estimates are heavily biased towards Europeans, probably limiting the performance of such PRSs in other populations or individuals with admixed ancestry. A recent genome-wide PRS to predict CKD trans-ethnically observed significant differences in the mean and variance of the PRS distributions by ancestry, mainly driven by a higher average of risk allele frequencies in some groups [53]. Although this is particularly relevant for underrepresented groups, such as populations with African and Latin American ancestry, Hispanic people, and native or indigenous groups [54,55,56,57], it may have had some impact on the lack of association for the SNPs and PRSs investigated in our cohort. In an attempt to avoid this issue and to have a sample whose ethnicity was internally homogeneous but similar to many reported studies, we selected only those participants of the UKB project within the European ancestry genetic ethnic group [58]. Beyond ancestry, the genetic data from which PRSs are derived come from generally healthier, higher socioeconomic groups [59] and therefore may exacerbate health inequalities, particularly for complex traits [60], such as kidney disease. In the UKB data, it has recently been demonstrated that even within a single ancestry group, the predictive accuracy of PRSs can be influenced by sociodemographic characteristics such as the age or sex of the individuals in which the GWAS and the prediction were conducted and by the GWAS study design itself [60]. DKD has shown sexual dimorphism or differences in prevalence, severity, or presentation between males and females [61]. After albuminuria, the male gender is the second most important risk factor for incipient or overt DKD [62]. Men have a faster progression from DKD to CKD and ESRD than premenopausal women [63,64] and have higher albuminuria and lower eGFR levels [62,65,66]. In this regard, the role of sex-specific effects and the impact on prediction performance of PRSs derived from the ratio of males to females in the sample used would be better explored with sex-stratified GWAS approaches and the integration of information from different biobanks as a meta-analysis [67]. The approach used to create the PRSs (imputed vs. genotyped SNPs, *p*-value threshold for inclusion, whether and how to account for LD) [57] may also affect the accuracy of the PRSs, as demonstrated using the Health and Retirement Study [68].

This study was cross-sectional and referred only to prevalent albuminuria; therefore, no incident cases were explored, which is a limitation of this study design [69]. The diabetic cohort of the UKB was less than 10% of the overall cohort and therefore had lower power and precision to detect associations for less common SNPs, which may have limited the detection of associations in this cohort without introducing any bias [70]. To prevent further reductions in the effective sample size with relevant covariates available, the duration of diabetes (available only for less than 50% of the participants) was not included as a covariate in the analysis of the diabetic cohort. Furthermore, our definition of diabetes included self-assessment, serum glycated hemoglobin, and fasting blood glucose, as well as indications of diabetes in the variables participant operations (Data Field #20004) and non-cancer illness (Data Field #20002), as described in Supplementary Table S1, along with other medications (Data Field #20003) to minimize the misclassification of diabetic patients; ICD-10 codes were not available. However, some more recent treatments for diabetes did not appear in this field; therefore, we were restricted to the drugs recorded in UKB participants.

## 4. Methods and Materials

### 4.1. Study Design and Population

This was a cross-sectional study utilizing UKB data. The UKB is the largest population-based cohort recruited in the United Kingdom to collect genetic and phenotypic information from approximately 500,000 individuals between 40 and 69 years old [58]. We selected only individuals classified by the UKB within the European ancestry genetic ethnic group (Data Field #22001) [58]. 

Individuals were excluded if they withdrew consent; inferred sex did not match reported sex; kinship was not inferred; putative sex chromosome aneuploidy; excessive heterozygosity or missingness; second degree and over-relatedness (KING coefficient > 0.0884); non-European ancestry (based on centralized sample quality control performed by UKB) [58]. 

The influence of the gene variants on the different phenotypic outcomes was investigated with and without stratification by diabetes. The total (Overall) cohort (389,206 participants) was therefore subdivided according to the presence (DM, 35,806 participants) or absence of diabetes mellitus (nonDM, 353,400 participants). 

### 4.2. Phenotypic Variables

#### 4.2.1. Outcome Variables

##### Kidney Damage 

Kidney damage was defined by the presence of any pathology, condition or medication indicative of renal injury, according to the UKB information provided in the variables non-cancer illness (Data Field #20002), participants operations (Data Field #20004) and medication (Data Field #20003). Details can be found in Supplementary Table S1.

##### Macroalbuminuria

Macroalbuminuria was defined as urine albumin-to-creatinine ratio (UACR) over 300 mg/g, i.e., the A3 category defined by the KDIGO guidelines (severely increased albuminuria) [8].

##### Microalbuminuria

Two definitions of microalbuminuria (mAlb) were analyzed, one sex-independent, defined as UACR over 30 mg/g i.e., the A2 category defined by the KDIGO guidelines (moderately increased albuminuria) [8], and one sex-specific, considered positive if UACR > 25 mg/g in women and >17 mg/g in men [71,72].

##### UACR

Urinary albumin values below the detection limit of the used assays were set to the lower limit of detection. The UACR was calculated as urinary albumin/urinary creatinine (mg/g) to account for differences in urine concentration. 

#### 4.2.2. Independent Variables (Confounders)

##### Anti-Hypertensive Medication

Participants were considered to have treated hypertension if they had any record of anti-hypertensive drugs falling into the following categories: angiotensin-converting enzyme inhibitors (ACEi), angiotensin II receptor blockers (ARBs), diuretics, calcium channel blockers, beta-blockers, alpha-blockers, and combination drugs from these categories, identified in UKB Data Field #20003 (treatment/medication code). Anti-platelet drugs, anticoagulants, anti-angina medications, anti-arrhythmic agents, statins, and medicines for erectile dysfunction or prostate enlargement (which have hypotensive actions) were excluded since they are not being used directly for the management of hypertension. Participants were also considered under anti-hypertensive medication if they had the category “blood pressure medication” in Data Fields #6177 (Medication for cholesterol, blood pressure, or diabetes) or #6153 (Medication for cholesterol, blood pressure, diabetes, or take exogenous hormones).

##### Cholesterol Medication

Participants were considered to be in receipt of cholesterol-lowering medication if they were assigned to the category “cholesterol-lowering medication” in UKB Data Fields #6177 (Medication for cholesterol, blood pressure, or diabetes) or #6153 (Medication for cholesterol, blood pressure, or diabetes, or take exogenous hormones), or had the treatment or medication codes 1140861958 (simvastatin), 1140888594 (fluvastatin), 1140888648 (pravastatin), 1140910632 (eptastatin), 1140910654 (velastatin), 1141146234 (atorvastatin), or 1141192410 (rosuvastatin) in Data Field #20003.

##### Diabetes

Participants were considered to have diabetes (either type 1 or type 2) if their glycated hemoglobin A1c was 48 mmol/mol (Data Field #30750) or had a blood glucose of 7 mmol/L after fasting for >8 h (Data Fields #30740, Glucose, and #74, Fasting Time); individuals whose diabetes was diagnosed by a doctor (Data Field #2443) were also included in the analysis. Other medications (Data Field #20003), non-cancer illnesses (Data Field #20002), and participant operations (Data Field #20004) were also considered to define diabetes. Details can be found in Supplementary Table S1.

##### Insulin

Participants were considered to be receiving insulin treatment when they had the category “Insulin” in UKB Data Fields #6177 (medication for cholesterol, blood pressure, or diabetes) or #6153 (medication for cholesterol, blood pressure, diabetes, or taking exogenous hormones) or had the treatment or medication code 1140883066 (insulin product) in Data Field #20003.

##### Other

Other clinical variables were age (Data Field #21003), sex (Data Field #22001), body mass index (Data Field #21001, kg/m^2^), and ever smoking (Data Field #20116, never vs. previous or current).

### 4.3. Statistical Analysis

#### 4.3.1. Descriptive Analysis 

Descriptive analysis was performed using R version 4.2.2 [73]. Qualitative variables were expressed as absolute values and percentages (%). Quantitative variables were expressed as mean and standard deviation or median and interquartile range (Q1–Q3) depending on normality. Normality was assessed with the Kolmogorov–Smirnov test. 

#### 4.3.2. Bivariate Analysis

Bivariate analysis was performed for all outcomes and covariates using the t-student test, the Mann–Whitney–Wilcoxon test, the chi-squared test with Yates’s correction for continuity, or the Spearman correlation tests, according to the nature of the variables. Variables associated with the bivariate analysis were then taken into the multivariate models (*p* < 0.05). Clinical variables with *p* > 0.05 in one cohort were maintained for the homogeneity of the models in the multivariate analysis.

#### 4.3.3. Genotyping, Imputation, and Quality Control

The Applied Biosystems^TM^ UKB Axiom^TM^ and UK BiLEVE Axiom^TM^ Affymetrix (now part of Thermo Fisher Scientific, Waltham, MA, USA) Arrays were used for genotyping by the UKB. Genotypes were imputed by the UKB using a combination of the Haplotype Reference Consortium and merged UK10K and 1000 Genomes phase 3 reference panels [58]. PLINK 1.90 beta and PLINK 2.00 alpha were used to perform quality control and extraction of dosages [74,75]. Related individuals (identity by kinship coefficient >0.0884) and principal component analysis (PCA) outliers, along with those with a high missingness rate or call rate lower than 95% as calculated by the UKB, were also removed [58]. SNPs were included in the study when they had been previously identified in GWAS as associated with microalbuminuria (sex-specific or KDIGO definition), UACR, or AER for individuals of European ancestry, not performed on UKB participants [25,26,27,28,29,32,33]. From studies that reported several SNPs within the same linkage disequilibrium (LD) block, the variant with the lowest *p*-value was selected [26]. Variants with minor allele frequency (MAF) <1%, or which were in LD with another variant (r^2^ > 0.7) were removed from the analysis. No values of R2 over 0.1 were found for the remaining set of variants, except for the pairs rs12509729-rs1564939 (R2 = 0.63) and rs1801239-rs10795433 (R2 = 0.38), which were not used simultaneously in either multivariable models or calculation of the PRS. SNPs not fulfilling Hardy–Weinberg Equilibrium (HWE, *p* < 0.05) or having an imputation score under 0.3 were also excluded. 

#### 4.3.4. Genetic Risk Score Derivation

The weighted polygenic risk scores (PRS) for microalbuminuria [25,26,27,28] and UACR [25,26,27,28,29,32,33] were calculated for each participant using the dosage of the SNPs identified in the different studies and their originally reported beta coefficients in Stata [76]. Studies using the UKB as a data source were excluded to avoid the overrepresentation of SNPs and/or individuals [37]. The PRS for microalbuminuria was composed of 29 SNPs, and 28 were included in the PRS to predict UACR. Specific variants and summary statistics used to calculate the PRSs are detailed in Supplementary Table S2, along with a description of the original GWAS. 

#### 4.3.5. Multivariable Analysis

Multivariable logistic regression was used to evaluate the genetic effects of SNPs or PRS on microalbuminuria, macroalbuminuria, and kidney damage, and linear regression for the natural logarithmically transformed UACR with/without stratification by diabetes (the threshold for the association: *p*-value < 0.05), using R version 4.2.2 [73]. Covariates included in the analysis were age, sex, body mass index, anti-hypertensive medication, cholesterol medication, and ever-smoking, along with diabetes and insulin for the overall strata. The kidney damage phenotype models were not adjusted by anti-hypertensive medication since this variable was part of the outcome definition. Ninety-one SNPs, considered additive genetic models, were investigated (Supplementary Table S3). Collinearity statistics (tolerance and variance inflation factors) and R square were calculated for linear regression, and regression assumptions were assessed with residual plots. McFadden’s R squared was calculated for logistic regression. Different multivariable models were constructed to assess separately either SNPs or PRS for both microalbuminuria and UACR. A backward stepwise approach was used, selecting variables with *p*-value < 0.05 to find a reduced explanatory model. The significance of each overall SNP and PRS model was compared to their corresponding clinical model (including only clinical and sociodemographic variables) using a Chi-square test.

#### 4.3.6. ROC Analysis

To test the discriminative ability of the different models (PRS or multivariable analysis equation), the receiver operating characteristic (ROC) curve and the area under the ROC curve (AUROC) was calculated using Stata [76]. ROC curves were compared using DeLong’s test for two correlated ROC curves, calculated with the pROC package [77] in R version 4.2.2 [73].

Integrated discrimination improvement (IDI) and net reclassification improvement (NRI) indices [78] were calculated to determine the clinical utility of the addition of SNPs combined into a PRS. The PRS was added to the model containing clinical and sociodemographic risk factors (the clinical model). Calculations were performed using the Hmisc package, version 4.7-2 [79], in R version 4.2.2 [73]. 

#### 4.3.7. Power Calculations 

Power calculations were performed for dichotomous traits considering the prevalence of microalbuminuria (sex definition) in each cohort and based on the minimum and maximum MAF of the variants included (rs142823282: 0.0116 and rs13079877: 0.4957, respectively), an allelic odds ratio (OR) of 1.2, and α = 0.05, for each cohort. The power to detect the variant with the minimum MAF was 100% both in the overall/non-diabetic cohorts and 55% in the cohort with diabetes. The power to detect the variant with the maximum MAF (rs13079877: 0.4957) was 100% for all cohorts.

## 5. Conclusions

Approximately half of the genetic variants investigated (41/91; 45%) demonstrated associations with the renal phenotypes in the UKB cohort, providing further evidence that these variants are associated with renal injury in European ancestry populations. Furthermore, more than half of these SNPs (22/41; 54%) displayed consistent directions of effect among the same or similar renal phenotypes. 

Although most of the previously reported genetic variants associated with albuminuria (microalbuminuria, macroalbuminuria, UACR, or AER) are consistently replicated in our study, their use as additional tools for improving clinical risk prediction is not justified. The genetic variants associated with albuminuria, either as individual SNPs or PRSs, did not improve the overall prediction of albuminuria or renal injury already given by clinical data. Enthusiasm for the use of PRS should be tempered by its limitations in predicting disease with multiple modifiable risk factors [80].

### Future Perspectives

The use of either individual SNPs or PRSs to predict albuminuria in renal disease is challenging given the very small effect sizes of individual risk alleles. The specificity of most of the genetic variants for certain ethnicities may also limit their extrapolation to other populations, reducing the generic applicability of identified risk alleles. In the future, a more comprehensive integration of multi-omic biomarkers might be feasible to improve the prediction of clinical phenotypes and provide clinicians with tools to improve the precision of individualized treatment plans. 

## Figures and Tables

**Table 1:** Descriptive analysis of the participants included in the study, stratified by diabetes. KDIGO: Kidney Disease: Improving Global Outcomes guidelines. N: Total number. n (%): Frequency (percentage). sd: Standard Deviation. UACR: urine albumin-to-creatinine ratio. Quantitative variables expressed as mean and standard deviation or median and interquartile range. Qualitative variables expressed as frequency and percentages.

	Overall Cohort		Non-Diabetic Cohort		Diabetic Cohort	
Variable	N	n (%) Mean ± sd|P_50_ [P_25_-P_75_]	N	n (%) Mean ± sd|P_50_ [P_25_-P_75_]	N	n (%) Mean ± sd|P_50_ [P_25_-P_75_]
Age (years)	389,206	56.9 ± 8.0	353,400	56.7 ± 8.0	35,806	58.6 ± 7.6
Sex (male)	389,206	179,240 (46.1)	353,400	159,874 (45.2)	35,806	19,366 (54.1)
Body Mass Index (kg/m^2^)	389,206	27.4 ± 4.7	353,400	27.2 ± 4.6	35,806	29.4 ± 5.7
Diabetes (yes)	389,206	35,806 (9.2)	353,400	0 (0)	35,806	35,806 (100)
Ever Smoker (yes)	389,206	176,499 (45.4)	353,400	158,263 (44.8)	35,806	18,236 (50.9)
Hypertensive Medication (yes)	389,172	91,381 (23.5)	353,374	74,674 (21.1)	35,798	16,707 (46.7)
Cholesterol Medication (yes)	389,206	69,617 (17.9)	353,400	52,285 (14.8)	35,806	17,332 (48.4)
Insulin (yes)	389,206	3931 (1.0)	351,942	0 (0)	35,522	3931 (11.1)
Microalbuminuria | sex-specific (yes)	389,206	56,682 (14.6)	353,400	49,639 (14.1)	35,806	7043 (19.7)
Microalbuminuria | KDIGO (yes)	389,206	29,260 (7.5)	353,400	25,161 (7.1)	35,806	4099 (11.5)
Macroalbuminuria (yes)	389,206	1429 (0.4)	353,400	1017 (0.3)	35,806	412 (1.2)
Kidney Damage (yes)	368,108	56,279 (15.3)	333,316	43,763 (13.1)	34,792	12,516 (36.0)
UACR (mg/g)	389,206	9.8 [6.1–16.5]	353,400	9.7 [6.1–16.3]	35,806	10.3 [6.4–17.9]

## Data Availability

The primary data used and/or analyzed during the current study are available from the UKB. This research was conducted using the UK Biobank Resource under Application Number 14259. Detailed phenotyping information, the polygenic risk score and summative data are presented in this paper.

## Supplementary Materials

The following supporting information can be downloaded at: https://www.mdpi.com/article/10.3390/ijms241311209/s1**.**

## Abbreviations

ACEi	angiotensin-converting enzyme inhibitors
AER	albumin excretion rate
AUROC	area under the receiver operating characteristic curve
BMI	body mass index
CKD	chronic kidney disease
CKD-EPI	Chronic Kidney Disease Epidemiology Collaboration
DKD	diabetic kidney disease
DM	diabetes mellitus
eGFR	estimated glomerular filtration rate
ESRD	end-stage renal disease
GFR	glomerular filtration rate
GTEx	gene tissue expression
GWAS	genome-wide association studies
HWE	Hardy-Weinberg equilibrium
LD	linkage disequilibrium
KDIGO	Kidney Disease: Improving Global Outcomes
MAF	minor allele frequency
mAlb	microalbuminuria
PCA	principal component analysis
PRS	polygenic risk scores
ROC	receiver operating characteristic curve
QC	quality control
SNP	single nucleotide polymorphism
UACR	urine albumin-to-creatinine ratio
UKB	UK Biobank
