# Supplementary material for: Albuminuria-Related Genetic Biomarkers: Replication and Predictive Evaluation in Individuals with and without Diabetes from the UK Biobank
Source: Int J Mol Sci. 2023 Jul 7;24(13):11209. doi: 10.3390/ijms241311209 (PMC10342310; doi:10.3390/ijms241311209)
Supplement: Supplementary file 1 [file ijms-24-11209-s001.zip › ijms-2454227-figures.pdf]

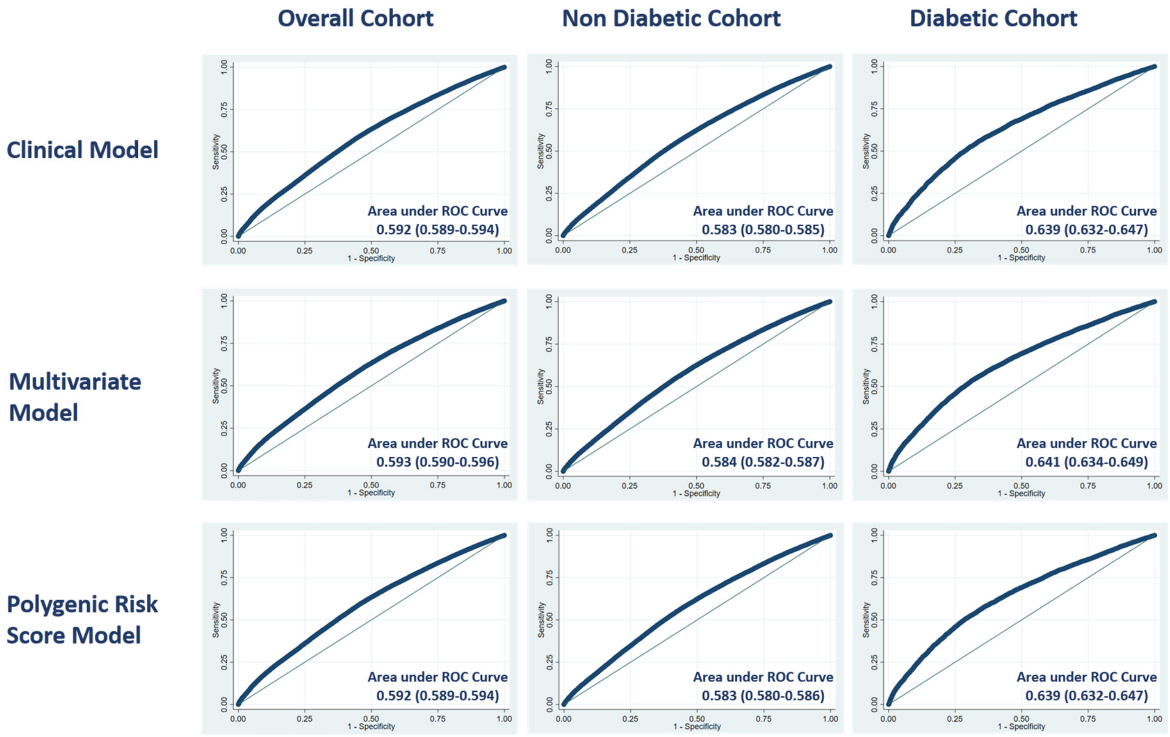

Supplementary Figure S1. Receiver operating characteristic (ROC) curve and area under the curve (AUROC) for the clinical, multivariable and polygenic risk score models for microalbuminuria (sex-specific definition) in the overall, non-diabetic and diabetic cohorts of the UK Biobank.

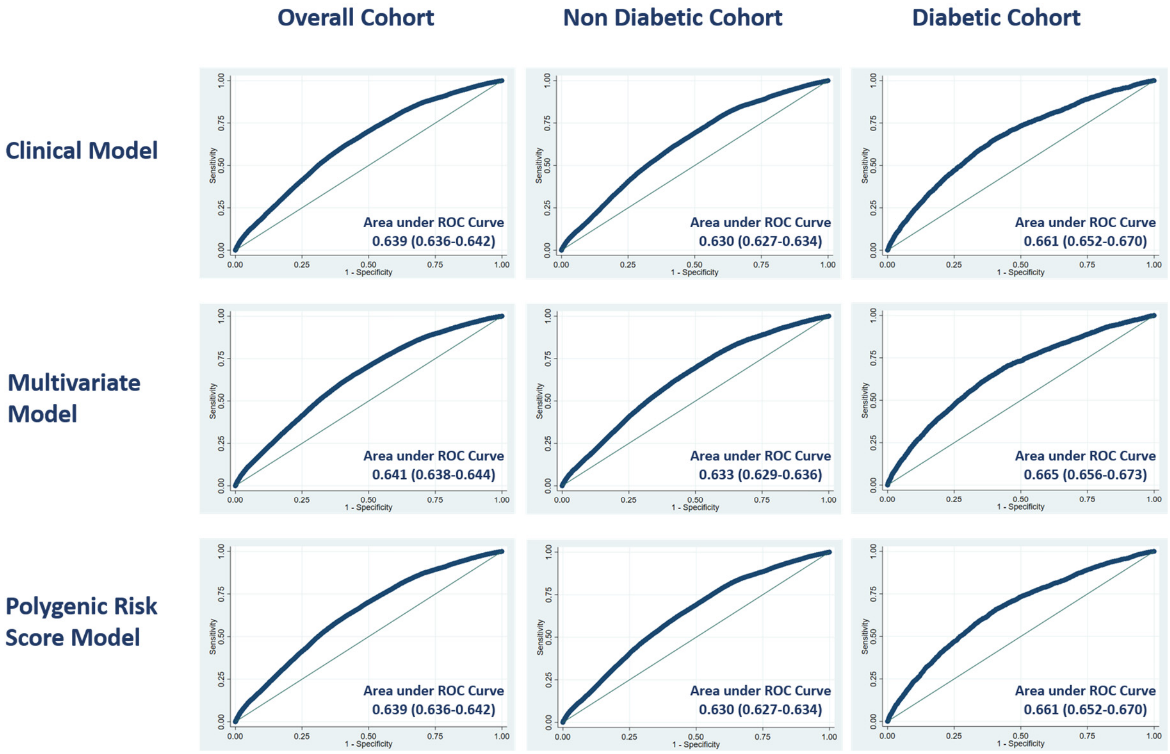

Supplementary Figure S2. Receiver operating characteristic (ROC) curve and area under the curve (AUROC) for the clinical, multivariable and polygenic risk score models for microalbuminuria (KDIGO definition) in the overall, non-diabetic and diabetic cohorts of the UK Biobank. KDIGO: Kidney Disease: Improving Global Outcomes.

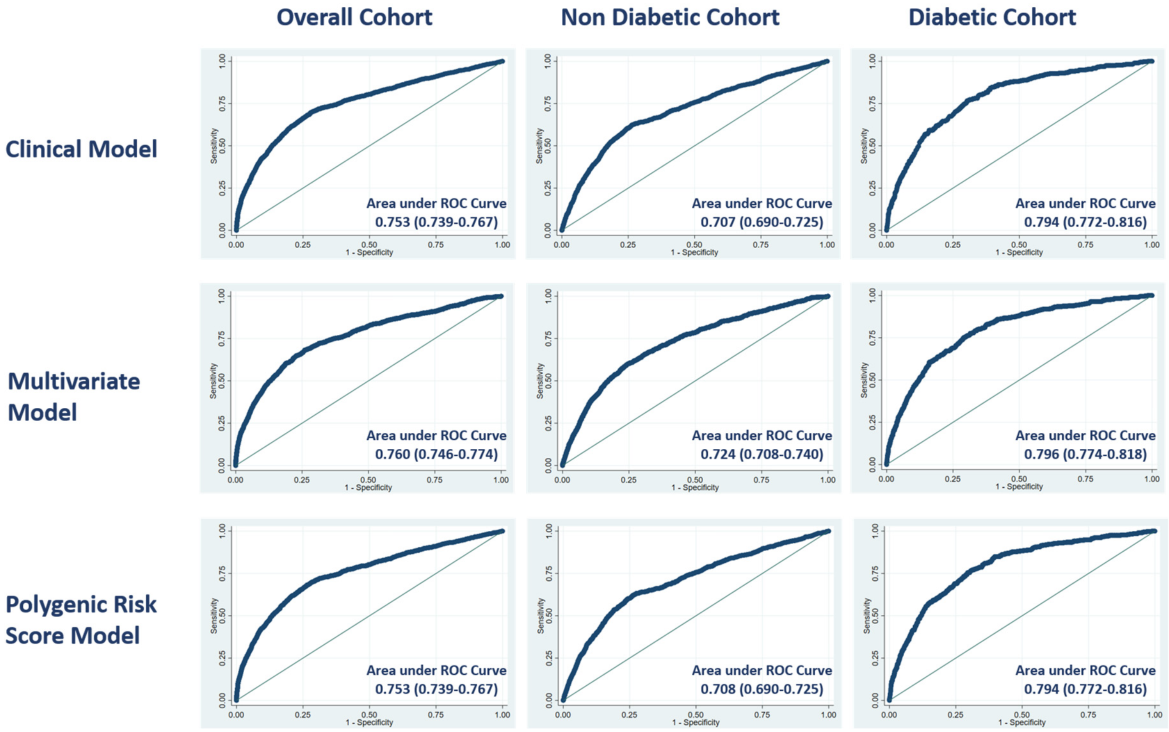

Supplementary Figure S3. Receiver operating characteristic (ROC) curve and area under the curve (AUROC) for the clinical, multivariable and polygenic risk score models for macroalbuminuria in the overall, non-diabetic and diabetic cohorts of the UK Biobank.

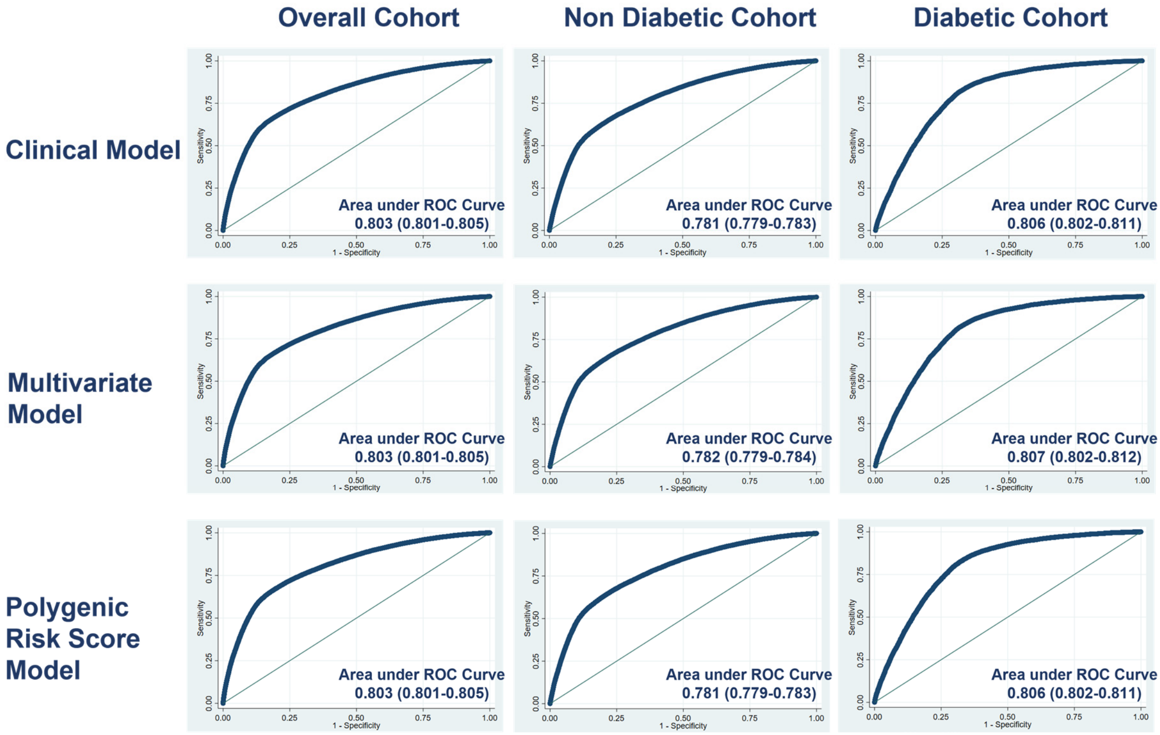

Supplementary Figure S4. Receiver operating characteristic (ROC) curve and area under the curve (AUROC) for the clinical, multivariable and polygenic risk score models for kidney damage in the overall, non-diabetic and diabetic cohorts of the UK Biobank.
